# Transversus abdominis plane block versus perioperative intravenous lidocaine versus patient-controlled intravenous morphine for postoperative pain control after laparoscopic colorectal surgery: study protocol for a prospective, randomized, double-blind controlled clinical trial

**DOI:** 10.1186/1745-6215-15-476

**Published:** 2014-12-04

**Authors:** Geertrui Dewinter, Marc Van de Velde, Steffen Fieuws, Andre D’Hoore, Steffen Rex

**Affiliations:** Department of Anaesthesiology, KU Leuven - University of Leuven, University Hospitals of Leuven, B-3000 Leuven, Belgium; Department of Cardiovascular Sciences, KU Leuven - University of Leuven, B-3000 Leuven, Belgium; I-Biostat, KU Leuven - University of Leuven, B-3000 Leuven, Belgium; Department of Abdominal Surgery, KU Leuven - University of Leuven, University Hospitals of Leuven, B-3000 Leuven, Belgium

**Keywords:** Colorectal surgery, Postoperative pain

## Abstract

**Background:**

Despite the laparoscopic approach becoming the standard in colorectal surgery, postoperative pain management for minimally invasive surgery is still mainly based on strategies that have been established for open surgical procedures. Patient-controlled epidural and intravenous analgesia are considered standard postoperative analgesia regimens in colorectal surgery. Epidural analgesia provides excellent analgesia, but is increasingly scrutinized in laparoscopic surgery since postoperative pain after the laparoscopic approach is significantly reduced. Moreover, epidural analgesia can be associated with numerous complications. Therefore, epidural analgesia is no longer recommended for the management of postoperative pain in laparoscopic colorectal surgery. Likewise, patient-controlled intravenous analgesia is subject to significant side effects. Given these important limitations of the traditional strategies for postoperative analgesia, effective and efficient alternatives in patients undergoing laparoscopic colorectal surgery are needed. Both the transversus abdominis plane block and systemically administered lidocaine have already been reported to effectively reduce pain after laparoscopic colorectal surgery. We hypothesize that the transversus abdominis plane block is superior to perioperative intravenous lidocaine.

**Methods/design:**

One hundred and twenty five patients undergoing laparoscopic colorectal surgery will be included in this prospective, randomized, double-blind controlled clinical trial. Patients will be randomly allocated to three different postoperative strategies: postoperative patient-controlled intravenous analgesia with morphine (control group, n = 25), a transversus abdominis plane block with ropivacaine 0.375% at the end of surgery plus postoperative patient-controlled intravenous analgesia with morphine (TAP group, n = 50), or perioperative intravenous lidocaine plus postoperative patient-controlled intravenous analgesia with morphine (LIDO group, n = 50). As the primary outcome parameter, we will evaluate the opioid consumption during the first 24 postoperative hours. Secondary endpoints include the Numeric Rating Scale, time to return of intestinal function, time to mobilization, inflammatory response, incidence of postoperative nausea and vomiting, length of hospital stay and postoperative morbidity as assessed with the Clavien-Dindo classification.

**Discussion:**

Recognizing the importance of a multimodal approach for perioperative pain management, we aim to investigate whether a transversus abdominis plane block delivers superior pain control in comparison to perioperative intravenous lidocaine and patient-controlled intravenous analgesia with morphine alone.

**Trial registration:**

EudraCT Identifier: 2014-001499-73; 31 July 2014.

## Background

A laparoscopic approach is now considered the gold standard in colorectal resection for benign and malignant disease [1]. Laparoscopic surgery is associated with a significant reduction in postoperative pain and opioid consumption, lower morbidity, faster recovery and shorter hospital stay [1]. However, strategies for postoperative pain management after laparoscopic surgery are mainly derived from concepts that have been established for open surgical procedures [2]. As such, patient-controlled epidural and intravenous analgesia are still the most frequently used techniques for postoperative analgesia after laparoscopic colorectal surgery [3]. Epidural analgesia (EA) is known to provide excellent pain control; however, the role of EA in laparoscopic surgery is increasingly being scrutinized [1]. Following laparoscopic colorectal surgery, the use of EA has been shown to result in a prolonged time to mobilization, an increase in hospital costs, length of hospital stay and a higher incidence of urinary tract infections [1, 4] EA can also be associated with disastrous complications including epidural hematoma or abscess [5]. An additional problem is that central neuraxial anesthesia cannot be applied in patients with chronic anticoagulant therapy or those suffering from coagulopathies.

Modern multimodal analgesia concepts have been demonstrated to provide postoperative analgesia as equally as effective as EA in patients undergoing laparoscopic colorectal surgery [3, 6]. Therefore, recently published guidelines from the UK do no longer recommend EA as standard therapy for pain control after laparoscopic colorectal surgery [1, 7].

The majority of multimodal analgesia concepts rely on the systemic administration of opioids under the control of the patient (patient-controlled intravenous analgesia (PCIA)). Unfortunately, PCIA with morphine is frequently limited by important side effects including sedation, constipation, itching, postoperative nausea and vomiting (PONV), and respiratory depression [8].

There is a quest for effective and efficient alternatives for postoperative analgesia in patients undergoing laparoscopic colorectal surgery.

The transversus abdominis plane (TAP) block is a relatively new regional anesthesia technique that provides analgesia of the parietal peritoneum, the anterior abdominal wall, and the skin [9]. Performed under ultrasound guidance, the block has been demonstrated to be simple and safe. [9] Furthermore, the use of TAP blocks results in a reduced cumulative opioid consumption in the first 24 postoperative hours [10].

In the last years, the use of systemic lidocaine as a co-analgesic has gained increasing interest for the treatment of acute postoperative pain. Lidocaine is a local anesthetic amide with analgesic, antihyperalgesic and anti-inflammatory properties [11]. Published data on the efficacy of the systemic administration of lidocaine perioperatively are inconsistent [11]. In abdominal surgery, lidocaine has been demonstrated to result in lower postoperative pain scores, a significantly reduced use of anesthetics and of postoperative analgesics [12, 13].

While both the TAP block and systemically administered lidocaine have been reported to effectively reduce pain after laparoscopic colorectal surgery, no randomized data comparing the role of TAP block versus systemic lidocaine are available [10, 14–16].

Given the fact that the TAP block is a potent locoregional anesthesia technique, we hypothesize that, in patients undergoing laparoscopic colorectal surgery, TAP block provides superior postoperative analgesia when compared to perioperative intravenous lidocaine.

## Methods/design

### Study design

**Figure 1:**
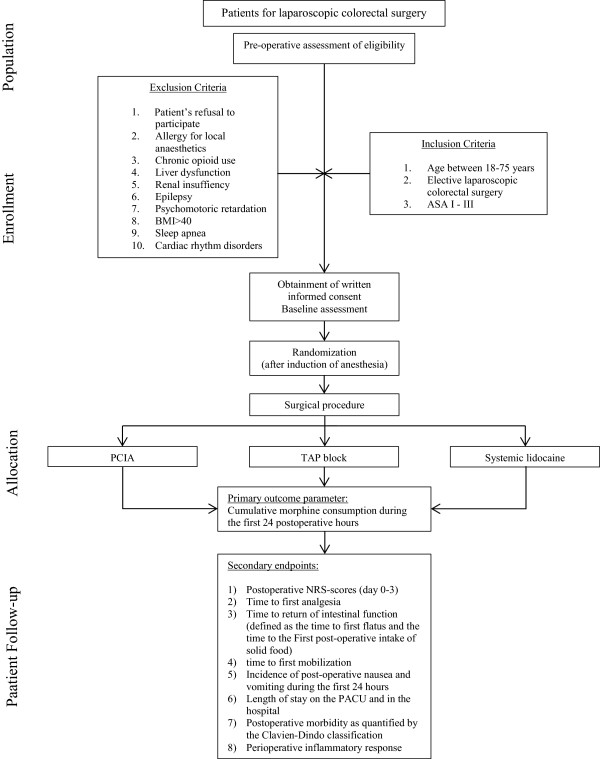
**Trial design chart.** ASA, American Society of Anaesthesiologists; NRS, numeric rating scale; PACU, postoperative anesthesia care unit; PCIA, patient-controlled intravenous analgesia; TAP, transversus abdominis plane.

This study is a single-center, prospective, randomized, double-blind, controlled trial. The study will be performed in accordance with the Declaration of Helsinki and has been approved by the ethics committee of the University Hospitals of the KU Leuven on 31 July 2014 with the reference number ML10699. The trial is registered under EudraCT 2014-001499-73. Figure 1 provides an overview of the trial design.

### Population

All consecutive patients scheduled for elective laparoscopic resection and candidates for enhanced recovery after surgery will be included in the study after informed consent has been obtained. Patients will be recruited in the Department of Abdominal Surgery of the University Hospitals of the KU Leuven.

### Randomization

Patients will be randomized to one of three groups that will either receive a TAP block with ropivacaine 0.375% at the end of surgery plus postoperative PCIA with morphine (TAP group, n = 50), perioperative intravenous lidocaine plus postoperative PCIA with morphine (LIDO group, n = 50), or only postoperative PCIA with morphine (control group, n = 25). The patients will be randomized to one of the study groups using a computer generated list. Allocation concealment will be ensured by enclosing assignments in sealed, opaque, sequentially numbered envelopes which will be opened only upon arrival of the patient in the operation room [17].

Postoperative outcomes will be assessed by research personnel that remain blinded to the type of intervention throughout the study.

### Inclusion and exclusion criteria

The inclusion criteria are: (1) aged between 18 and 75 years, (2) elective laparoscopic colorectal surgery, (3) American Society of Anesthesiologists risk classification < IV.

The exclusion criteria include: (1) refusal of the patient, (2) known hypersensitivity to study medications, (3) chronic opioid use, (4) liver insufficiency (defined as a serum bilirubin ≥2 mg/dl), (5) renal insufficiency (defined by estimated glomerular filtration rate ≤60 ml/min/1.73 m^2^), (6) epilepsy, (7) psychomotoric retardation, (8) morbid obesity (defined as a body mass index >40), (9) obstructive sleep apnea syndrome, (10) cardiac rhythm disorders.

### Intervention plan

In all patients, the Enhanced Recovery After Surgery protocol will be used in order to standardize perioperative treatment in all groups [18]. The Enhanced Recovery After Surgery protocol comprises: (1) no preoperative bowel preparation, (2) avoidance of prolonged fasting, (3) no premedication, (4) intraoperative administration of PONV and antibiotic prophylaxis, (5) maintenance of normothermia, (6) restrictive fluid management, (7) early postoperative removal of the gastric tube and the bladder catheter, (8) early oral nutrition, and (9) early mobilization of the patient.

#### Induction and maintenance of anesthesia

After preoxygenation, anesthesia will be induced with a bolus injection of propofol (2 mg/kg) and an intravenous infusion of remifentanil (target-controlled infusion with a calculated plasma level of 4 to 5 ng/ml). Tracheal intubation will be facilitated with cis-atracurium (0.15 mg/kg). General anesthesia will be maintained by inhalation of sevoflurane. In all groups, sevoflurane end-tidal concentrations will be titrated according to the instantaneously registered electroencephalography monitor in order to achieve a bispectral index value between 40 and 60.

PONV prophylaxis will be achieved with intravenous dexamethasone (0.1 mg/kg) at induction and intravenous ondansetron (0.1 mg/kg) 30 minutes before the end of surgery.

In all patients, standard American Society of Anesthesiologists monitoring will be applied, including electrocardiogram, pulse oximetry, capnography, and temperature measurements. In addition, patients will be monitored using bispectral index, invasive arterial and central venous pressure measurements, and relaxometry.

#### Interventional treatment

**TAP group** At the end of surgery, a bilateral single shot TAP block will be performed under ultrasound guidance. At each side, 20 ml ropivacaine 0.375% and clonidine 0.5 μg/kg will be injected into the “triangle of Petit”, which is located between the internal oblique muscle and the transverse abdominal muscle.

**LIDO group** A bolus of 1.5 mg/kg of intravenous lidocaine will be administered after induction of anesthesia, followed by a continuous infusion of intravenous lidocaine at 1.5 mg/kg per hour. The lidocaine infusion will be stopped 4 hours after arrival in the postoperative anesthesia care unit (PACU).

To achieve blinding of the patients and study observers, patients in the TAP group and in the control group will perioperatively receive a placebo infusion with saline at a comparable rate as the lidocaine infusion in the LIDO group. Moreover, in patients of both the LIDO group and the control group, adhesive tapes will be attached at the level of the assumed TAP block puncture site.

#### Postoperative analgesia

Irrespective of group allocation, all patients will receive a combination of intravenous analgesics 30 minutes before the end of the surgery: paracetamol 15 mg/kg, ketorolac 0.5 mg/kg (maximum 30 mg), 0.2 mg/kg morphine.

Moreover, each patient will receive PCIA with morphine. The PCIA pump will be programmed in an on-demand-only mode without a basal rate, allowing for a bolus injection of 1.5 mg every 7 minutes with a maximum of 30 mg every 4 hours. The PCIA pump will be stopped on the third postoperative day.

Once the PCIA pump is discontinued, piritramide 0.5 mg/kg and paracetamol 15 mg/kg will be offered upon request.

PONV will be treated with intravenous droperidol 0.625 mg (in the PACU) or intravenous ondansetron 4 mg (on the ward).

#### Postoperative care unit

The patients will be transferred to the PACU for continuous monitoring of vital signs. The Aldrete score will be recorded. In the PACU, severity of pain will be assessed at rest and during coughing by a numeric rating scale (0 = no pain, 10 = the worst imaginable pain). As soon as the numeric rating scale score exceeds 3, patients will be treated with 1 mg intravenous morphine until freedom from pain is achieved. Severity of pain will be monitored every 15 minutes during the first 2 hours of the PACU stay and hourly during the remaining PACU stay. Patients will stay at least 4 hours in the PACU. Patients will be discharged from the PACU only once the Aldrete score is 9, and once there is no evidence of pain and/or PONV.

#### Follow-up visits

Patients will be visited once daily throughout their hospital stay by research personnel.

### Primary endpoint

As the primary outcome parameter, we will evaluate the cumulative morphine consumption in the first 24 postoperative hours.

### Secondary endpoints

Secondary outcome parameters include: (1) numeric rating scale; (2) time to return of intestinal function (defined as the time to first flatus and the time to the first postoperative intake of solid food); (3) time to first mobilization; (4) the incidence of PONV during the first 24 hours, (5) length of stay on the PACU and in the hospital; (6) postoperative morbidity as quantified by the Clavien-Dindo classification [18]; (7) perioperative inflammatory response (as measured by serial serum levels of C-reactive protein, interleukin-6, and interleukin-10 - for this, serum samples (5 ml each) will be obtained from the arterial catheter or the central venous line: (1) after induction of anesthesia, (2) at the end of surgery, (3) 12 hours after induction, (4) 24 hours after induction, (5) day 1, (6) day 2 and (7) day 3.

### Assessment of safety

The interventional treatment will be administered to patients with standard hemodynamic monitoring in the setting of a fully equipped operation theatre. This enables immediate detection and treatment of adverse events. Administration of study drugs will be immediately stopped in cases where the study participant shows a relevant deterioration. Also, after leaving the operation room, all patients will be closely monitored for the occurrence of eventual (severe) adverse events, first on the PACU and later on the surgical ward. Moreover, the inclusion of each individual patient into the study is indicated in the electronic hospital information system and, hence, is visible to all physicians and nurses involved in the care of the patient. This facilitates reporting of (severe) adverse events to the principal investigator. The principal investigator will report suspected unexpected serious adverse reactions to the federal health authorities.

### Data analysis

All statistical analyses will be performed using SAS software version 9.2 (SAS Inc., Cary, NC, USA). All tests will be two-sided, and significance will be set at *P* < 0.05.

Two-sided *t* tests for the ratio of means will be used to compare the opium consumption between the TAP and LIDO and between the TAP and control group, respectively; 95% confidence intervals for the ratio will be reported. If the log-transformed data (natural logarithm) show departure from normality (based on the Shapiro–Wilk W-test statistic) a Mann–Whitney U-test will be used to verify the robustness of the conclusion. Proportions will be compared between groups with Fisher's exact tests, and Mann–Whitney U-tests will be used to compare the length of stay and the postoperative morbidity classification.

Time-to-event outcomes will be measured from end of surgery until the occurrence of the event. Patients will be censored if they do not experience the event at the time of the last follow-up. Of note, the number of censored events is expected to be negligible since the events of interest (mobilization and return of intestinal function) are a prerequisite for a patient’s discharge from the hospital. Kaplan-Meier estimates will be used to obtain the cumulative distribution curves for the event times and groups will be compared using the log-rank test. A linear model for longitudinal measurements with an unstructured covariance matrix will be used to evaluate the evolution of the inflammatory response. An analysis will be performed for each serum marker separately, after applying a transformation of the response if needed to obtain a symmetric distribution of the model residuals.

Completed case record forms will be reviewed by an investigator or a study nurse for completeness and correctness before digitalization and statistical analysis. At this time point, missing data will be identified and, if possible, drawn from source data and filled into the case record forms. Missing data not being found in the source data is not expected as all clinical data (including routine data) are mandatorily collected in the electronic hospital information system and outcomes of all abdominal surgical patients are routinely documented in detail according to the standards of the British “Enhanced Recovery Programme”. In any case, data will be analyzed according to the intention-to-treat principle.

### Sample size calculation

The study has been powered to detect the differences in primary outcome between the TAP and the LIDO group, and between the TAP and the control group. The coefficient of variation in postoperative morphine consumption was derived from reported standard deviation or interquartile range values in the literature [10, 19] and found to be in the range 0.19 to 0.73. Further, in an unpublished retrospective evaluation of 10 patients in our institution, we observed a coefficient of variation of 0.35. Hence, a coefficient of variation equal to 0.5 was assumed in the power calculation. Using a two-sided test for a ratio of means (with alpha = 5%), 44 patients per group are needed to show a 25% reduction in the 24 hour morphine consumption in the TAP versus LIDO group when a power of 80% is to be achieved. The assumption that perioperative lidocaine also yields a reduction of 25% compared to PCIA alone (control group) implies a ratio of 0.75^2^ = 0.5625 for TAP versus control. Recruiting 22 patients in the control group yields more than 99% power to detect this difference, with 44 patients included in the TAP group and using the same test. As such, 110 patients in total are needed. To compensate for possible drop-outs, we will include 125 patients in total. They will be randomized into the TAP group, the LIDO group and the PCIA group with weights equal to 2, 2 and 1.

Note that to enable confirmatory claims about both comparisons without inflating the type I error, a hierarchical closed test procedure is used (that is, both comparisons are tested on a 5% level) with the comparison of TAP versus control being tested only in case that the comparison of TAP versus LIDO is significant.

## Discussion

### Benefits

Recently, both TAP block and perioperative intravenous lidocaine have been shown to efficiently control postoperative pain in patients after laparoscopic colorectal surgery. Both techniques have been demonstrated to reduce postoperative opioid consumption, but no comparative data are available. We hypothesize that the use of TAP blocks will be superior for postoperative pain control when compared with the perioperative administration of intravenous lidocaine or PCIA. The TAP block has been proven to be safe and is technically easy to perform, particularly under ultrasound guidance.

Possible benefits of the TAP block include its ease of performance, proven effectiveness, the reduction of postoperative opioid consumption, and the fact that it can be performed even in patients under anticoagulant therapy or with coagulopathies. Moreover, systemic side effects as happens with patient-controlled EA and PCIA are avoided.

### Risks

Risks are mainly due to the systemic toxicity of local anesthetics. The intravenous use of lidocaine may lead to a close-related systemic toxicity that can affect the central nervous system (drowsiness, confusion, euphoria, double vision, seizures) and the cardiovascular system (hypotension, bradycardia, arrhythmias). However, the doses as suggested in our study have repeatedly been demonstrated to be safe and to result in plasma concentrations that are far below the level of toxicity (5 μg/ml) [14]. As a safety measure, patients with impaired lidocaine metabolism due to liver dysfunction are excluded from study participation. Moreover, patients are continuously monitored by electrocardiography during the administration of lidocaine.

The use of ropivacaine for the TAP block may also cause system toxicity. The incidence of systemic toxicity of local anesthetics after peripheral nerve blocks is estimated to be 1:1000, with mostly minor symptoms. Of note, ropivacaine has an excellent safety profile with only minor cardiac toxicity. Ropivacaine has therefore become a popular choice for high-dose, high-volume fascial plane blocks including the TAP block. Risk of inadvertent intravenous, bowel or nerve injection will be minimized by ultrasound guidance and needle aspiration prior to injection [20].

According to the recent guidelines of the American Society of Regional Anesthesia, patients will be continuously monitored with electrocardiogram, blood pressure measurement and pulse oximetry for at least 30 minutes after injection of ropivacaine [21].

### Trial status

Patient recruitment will start in September 2014. The predicted study completion date is December 2015.

## Abbreviations

EA: epidural analgesia
PACU: postoperative anesthesia care unit
PCIA: patient-controlled intravenous analgesia
PONV: postoperative nausea and vomiting
TAP: transversus abdominis plane.
